# Closing the Gap: Endoscopic Management of Post-Surgical Gastrointestinal Fistulas Using the Padlock Clip™—A Single-Center Experience

**DOI:** 10.3390/jcm15093452

**Published:** 2026-04-30

**Authors:** Gabriela Ivanov, Madalina Ilie, Gabriel Constantinescu, Christopher Pavel, Deniz Günșahin, Raluca-Ioana Dascalu, Mariana Mihaila, Mircea Bogdan Maciuceanu Zarnescu, Anca Monica Macovei-Oprescu

**Affiliations:** 1 Department of Gastroenterology, Clinical Emergency Hospital of Bucharest, 105402 Bucharest, Romania; ivanov.gabriela.98@gmail.com (G.I.); gabrielcostantinescu63@gmail.com (G.C.); christopher.pavel@gmail.com (C.P.); raluca-ioana.dascalu@rez.umfcd.ro (R.-I.D.); 2 Department 5, Gastroenterology, “Carol Davila” University of Medicine and Pharmacy, 050474 Bucharest, Romania; bmaciuceanu@gmail.com (M.B.M.Z.); anca.macovei@umfcd.ro (A.M.M.-O.); 3 Department of Internal Medicine, Fundeni Clinical Institute, 022328 Bucharest, Romania; m.mihaila14@gmail.com; 4 Department of Plastic Surgery and Reconstructive Microsurgery, Clinical Emergency Hospital of Bucharest, 105402 Bucharest, Romania; 5 Department of Gastroenterology, Emergency Clinical Hospital Prof. Dr. Agrippa Ionescu, 011356 Bucharest, Romania

**Keywords:** post-surgical fistula, Padlock Clip™, over-the-scope clip, sleeve gastrectomy, therapeutic endoscopy

## Abstract

**Background/Objectives**: Post-surgical gastrointestinal fistulas represent severe complications associated with significant morbidity and therapeutic challenges. Over-the-scope clipping systems have expanded endoscopic management options; however, data regarding the Padlock Clip™ in mixed populations of acute and chronic fistulas remain limited. This study aimed to evaluate the feasibility, safety, and clinical outcomes of the Padlock Clip™ in the endoscopic management of post-surgical gastrointestinal fistulas in a tertiary referral center. **Methods**: We conducted a retrospective single-center study including 28 adult patients treated with the Padlock Clip™ between January 2021 and December 2025. Technical success, clinical success, reintervention rates, and adverse events were assessed. Exploratory subgroup analyses were performed according to surgical etiology and fistula complexity. **Results**: Technical success was achieved in 25/28 patients (89.3%), and clinical success occurred in 23/28 cases (82.1%). Reintervention was required in seven patients (25.0%). Adverse events occurred in three patients (10.7%) and were limited to clip migration. The median follow-up duration was 6.5 months (range 3–32 months). Sleeve gastrectomy-related fistulas demonstrated lower technical success compared with non-sleeve cases (72.7% vs. 100%). Immediate technical success was associated with a trend toward improved clinical outcomes. Complex fistulas showed lower healing rates and higher reintervention frequency. **Conclusions**: The Padlock Clip™ is a feasible and safe option for endoscopic management of post-surgical gastrointestinal fistulas. Immediate technical success appeared to be associated with improved clinical outcomes and may represent a potential factor in achieving durable fistula closure. Complex fistulas and sleeve gastrectomy-related defects may require multimodal therapeutic approaches.

## 1. Introduction

Post-surgical gastrointestinal fistulas represent one of the most severe complications following digestive surgery. They represent abnormal communications between the gastrointestinal lumen and adjacent organs, cavities, or the skin, and are associated with substantial morbidity, prolonged hospitalization, increased risk of sepsis, malnutrition, electrolyte imbalance, and mortality. These fistulas usually result from anastomotic failure, staple-line dehiscence, ischemia, infection, or technical factors and may present as early postoperative leaks or evolve into chronic fistulas when healing fails and epithelialization of the tract occurs. Their management is complex and often requires a multidisciplinary approach involving surgeons, interventional radiologists, and therapeutic endoscopists [1,2,3].

Within this broad spectrum of postoperative complications, bariatric surgery (particularly laparoscopic sleeve gastrectomy) has drawn increasing attention. Sleeve gastrectomy is currently the most frequently performed bariatric procedure worldwide, and staple-line leaks and fistulas remain its most serious adverse events, with reported incidence ranging from 0% to 7%. These defects most commonly occur near the gastroesophageal junction, an area characterized by high intraluminal pressure, limited vascular supply, and constant mechanical stress, factors that predispose to persistence and progression toward chronic fistula formation [4,5,6,7].

Traditional management of post-surgical fistulas includes antibiotic therapy, nutritional support, drainage of associated collections, and surgical revision. However, reoperation in this setting is often technically challenging and associated with considerable morbidity and mortality, particularly in patients with ongoing infection, dense adhesions, or poor nutritional status. As a result, minimally invasive strategies have progressively gained importance in treatment algorithms [1,8,9].

Over the past two decades, therapeutic endoscopy has assumed a central role in the management of gastrointestinal leaks and fistulas. A wide range of endoscopic techniques has been described, including self-expanding metal stents, tissue sealants, endoscopic suturing, internal drainage with double-pigtail stents, endoscopic vacuum therapy, and mechanical clipping devices. The choice of endoscopic approach depends on several factors, including fistula size, location, chronicity, tissue viability, and the presence of associated collections or distal obstruction [10,11,12,13].

Mechanical clipping devices play an important role among endoscopic closure techniques. Through-the-scope clips are effective for small mucosal defects but have limited efficacy in larger or fibrotic fistulas. These limitations led to the development of over-the-scope clip (OTSC) systems capable of full-thickness tissue capture and stronger compression [1,14,15].

OTSC devices have been widely applied for the closure of perforations, leaks, and fistulas throughout the gastrointestinal tract, including esophageal, gastric, small-bowel, colonic, and rectal defects. While high technical and clinical success rates have been reported for acute perforations and early leaks, outcomes for chronic fistulas are more variable and often require multimodal endoscopic therapy. A recent multicenter study reported favorable outcomes using OTSC for postoperative anastomotic leaks and fistulas, supporting their role as part of contemporary endoscopic management strategies rather than as standalone solutions in all cases [3,11,12,13].

The Padlock Clip™ is a more recent addition to OTSC technology. Unlike conventional bear-claw OTSC systems, it consists of a circumferential nitinol ring with inward-directed prongs that provide 360-degree tissue compression. The device is mounted on the tip of the endoscope and does not occupy the working channel, allowing simultaneous use of grasping devices during deployment. This design aims to facilitate uniform tissue capture and closure, particularly in difficult anatomical locations [2,12].

Early clinical experience with the Padlock Clip™ has demonstrated feasibility in a variety of settings, including closure of perforations, post-procedural defects, and gastrointestinal fistulas in both upper and lower GI locations. Most available evidence consists of case reports and small series describing high technical success with acceptable safety profiles, although long-term outcome data remain limited [14,15,16].

Although endoscopic clipping devices are increasingly incorporated into routine clinical practice, available evidence remains largely descriptive and heterogeneous, particularly for mixed populations that include both acute leaks and chronic post-surgical fistulas across different gastrointestinal locations [17,18,19,20]. Additional single-center experiences may therefore help clarify practical outcomes, limitations, and the role of clipping devices within multimodal endoscopic management.

Accordingly, we present our single-center experience using the Padlock Clip™ for endoscopic management of post-surgical gastrointestinal fistulas. Our cohort includes a mixed population of acute and chronic fistulas, predominantly occurring after sleeve gastrectomy and involving both upper and lower gastrointestinal segments, with the aim of providing pragmatic data on feasibility, technical success, clinical outcomes, and adjunctive endoscopic strategies in a real-world setting.

## 2. Materials and Methods

### 2.1. Study Design and Setting

This was a retrospective, single-center observational study conducted at the Clinical Emergency Hospital of Bucharest, a tertiary referral center. We reviewed 28 consecutive adult patients with post-surgical gastrointestinal fistulas who underwent endoscopic treatment using the Padlock Clip™ (STERIS Endoscopy, Mentor, OH, USA) between January 2021 and December 2025. Although predefined variables were extracted in a structured manner, all clinical, procedural, and follow-up data were collected retrospectively from existing medical records and endoscopy reports. All procedures were performed using Olympus endoscopic systems (Olympus Corporation, Tokyo, Japan). All patients signed an informed consent before each procedure. This retrospective study was conducted in accordance with the Declaration of Helsinki and approved by the Ethics Committee of Clinical Emergency Hospital of Bucharest (approval number 7331/12 February 2026).

### 2.2. Patient Selection

All adult patients (≥18 years) who underwent endoscopic treatment of a post-surgical gastrointestinal fistula using the Padlock Clip™ during the study period were eligible for inclusion. A post-surgical gastrointestinal fistula was defined as an abnormal communication between the gastrointestinal lumen and adjacent structures occurring after surgical intervention, including both acute and chronic fistulas.

Patients were included if:

- The fistula was confirmed endoscopically and/or radiologically;
- Endoscopic closure using the Padlock Clip was attempted;
- Clinical, endoscopic, and procedural data were available for review.

Patients were excluded if:

- Endoscopic treatment was not attempted;
- Closure was performed exclusively with other endoscopic modalities without Padlock Clip deployment;
- Follow-up data were unavailable.

### 2.3. Evaluation of Fistula Characteristics

Fistulas were characterized using predefined variables extracted from electronic medical records, endoscopy reports, and imaging data. These variables included the type of fistula, when specified (e.g., esophagopleural, esophagoperitoneal, esophagobronchial, or other communications), and the anatomical location, categorized as esophagus, esophagogastric junction, stomach, duodenum, rectum, or other gastrointestinal sites. The estimated fistula size was assessed endoscopically and classified as <15 mm or ≥15 mm. Fistula size was estimated endoscopically using comparison with accessory diameters such as open biopsy forceps and distal attachment cap dimensions. Because endoscopic estimation of irregular defects is inherently approximate, some degree of measurement variability should be considered when interpreting size-based subgroup analyses. The 15 mm threshold was selected based on previously published endoscopic closure studies and practical OTSC-based treatment algorithms suggesting reduced efficacy of clipping techniques in larger defects and increased need for multimodal strategies beyond this size range, as described in contemporary reviews of endoscopic management of postoperative gastrointestinal leaks and fistulas [9,12].

Fistulas were classified as simple or complex according to predefined procedural and anatomical criteria. Complex fistulas were defined as those measuring ≥ 15 mm or requiring multimodal endoscopic closure strategies, including deployment of more than one Padlock Clip or adjunctive therapies. Fistulas not meeting these criteria were classified as simple.

The therapeutic context was also recorded, distinguishing between primary endoscopic closure and rescue therapy performed after prior unsuccessful interventions, such as stent placement or other endoscopic modalities.

Due to retrospective design and referral-based patient pathways, differentiation between acute and chronic fistulas relied on clinical presentation, prior treatment history, and endoscopic appearance (fibrotic/epithelialized margins) rather than strict time-based criteria, as interval from index surgery was inconsistently documented.

### 2.4. Surgical Background

For each patient, surgical history was recorded, including:

- Underlying indication for index surgery (obesity/bariatric surgery, gastrointestinal malignancy, or benign gastrointestinal disease);
- Primary surgical procedure, with sleeve gastrectomy representing the most frequent individual surgical procedure;
- Associated disease leading to surgery, as documented in the medical record.

### 2.5. Endoscopic Procedure

All procedures were performed by experienced therapeutic endoscopists using standard therapeutic endoscopes (Olympus, Evis Exera X1) under deep sedation or general anesthesia, according to anesthesiology assessment and institutional protocols.

A diagnostic endoscopy was initially performed to assess fistula location, size, tissue viability, and suitability for clip deployment. In selected cases, particularly chronic fistulas with fibrotic margins, tissue preparation was performed using mechanical abrasion or thermal coagulation to promote granulation tissue formation and facilitate clip anchoring.

The Padlock Clip system was mounted onto the distal tip of the endoscope. The fistulous opening was centered within the cap, and surrounding tissue was drawn into the chamber using suction. The clip was then deployed to achieve circumferential full-thickness compression and defect closure.

Adjunctive therapies, including self-expanding metal stent placement or additional clipping, were used in selected cases based on defect characteristics and clinical context.

### 2.6. Outcome Measures

Demographic data (age, sex), surgical indication, primary surgery, fistula characteristics, procedural details, adjunctive endoscopic therapies, outcomes, and adverse events were retrospectively extracted from electronic medical records and endoscopy reports and recorded in a dedicated database. Follow-up data were obtained from clinical records, imaging studies, and repeat endoscopic evaluations when available. Because of the retrospective design, follow-up duration varied among patients, and complete long-term follow-up was not available in all cases. Clinical outcomes were therefore assessed based on the most recent available clinical, endoscopic, or radiological evaluation for each patient. Follow-up duration was defined as the interval between Padlock Clip deployment and the most recent available clinical, endoscopic, or radiological evaluation. Clinical success was defined as sustained fistula closure without the need for surgical intervention regardless of whether additional endoscopic therapies were required during follow-up.

### 2.7. Statistical Analysis

Data were initially collected in Microsoft Excel 365 (Microsoft Corp., Redmond, WA, USA) and subsequently analyzed using IBM SPSS Statistics version 31.0.1.0 (IBM Corp., Armonk, NY, USA). Descriptive statistics were used to summarize baseline characteristics. Categorical variables are presented as counts and percentages, and continuous variables are presented as median and range. Exploratory subgroup analyses were conducted using Fisher’s exact test due to the small sample size. Odds ratios (ORs) with 95% confidence intervals (CIs) were calculated using contingency table analysis (Crosstabs procedure in SPSS). All tests were two-tailed, and a *p*-value < 0.05 was considered statistically significant. Given the limited sample size and exploratory nature of the analyses, subgroup comparisons were considered hypothesis-generating.

## 3. Results

### 3.1. Baseline Characteristics and Surgical Background

A total of 28 patients were included in the analysis. The median age was 49.5 years (range 28–76). The cohort consisted predominantly of women, with 17 female patients (60.7%) and 11 male patients (39.3%).

Regarding the underlying pathology leading to the index surgical procedure, malignant conditions were present in 10 patients (35.7%), while benign gastrointestinal conditions accounted for 18 cases (64.3%). Among benign conditions, morbid obesity represented the majority, affecting 11 patients (61.1% of benign cases).

Gastric surgery was performed in 16 patients (57.1%), including 11 sleeve gastrectomies (39.3% of the total cohort). Other surgical procedures included abscess drainage (*n* = 3, 10.7%), low anterior rectal resection (*n* = 2, 7.1%), pulmonary lobectomy (*n* = 2, 7.1%), and other procedures such as transduodenal excision of a duodenal neuroendocrine tumor with pyloroplasty, Heller myotomy, cholecystectomy, and cytoreductive surgery for ovarian cancer involving colorectal resection (*n* = 5, 17.6%) (Table 1).

### 3.2. Anatomical Distribution and Features of Fistulas

The majority of defects involved the upper gastrointestinal tract, accounting for 25 cases (89.3%), whereas 3 fistulas (10.7%) were located in the lower gastrointestinal tract.

Regarding anatomical distribution, the esophagus was the most frequent location, observed in 10 patients (35.7%), followed by the esophagogastric junction in 5 cases (17.9%). Additional upper gastrointestinal locations included the duodenum (*n* = 4, 14.3%), esophagojejunal anastomosis (*n* = 3, 10.3%), and the stomach (*n* = 3, 10.3%). Lower gastrointestinal involvement included rectal fistulas in three patients (10.3%).

Most fistulas were small, with 26 defects (92.9%) measuring < 15 mm, while only two cases (7.1%) exceeded 15 mm in diameter.

**Figure 1:**
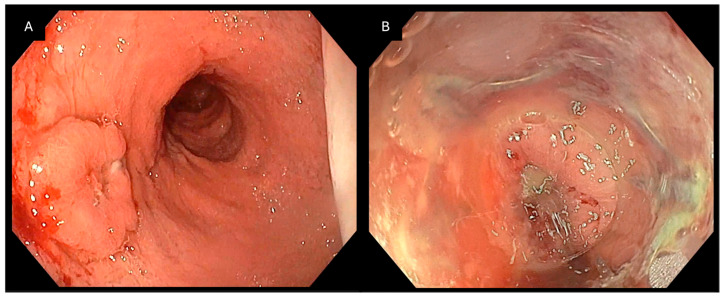
Endoscopic management of an esophagopleural fistula following sleeve gastrectomy: (**A**) Endoscopic view of a fistulous orifice located in the distal esophagus, consistent with an esophagopleural communication after sleeve gastrectomy. The defect is surrounded by inflamed mucosa. (**B**) Deployment of the Padlock Clip with circumferential tissue capture. Purulent drainage is visible at the time of closure, indicating active infection.

**Figure 2:**
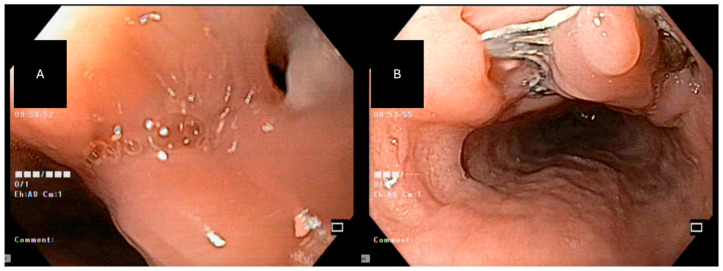
Endoscopic closure of an esophagobronchial fistula located in the mid-esophagus following pulmonary lobectomy for lung cancer: (**A**) Endoscopic visualization of a fistulous orifice in the mid-esophagus consistent with communication to the bronchial tree. (**B**) Deployment of the Padlock Clip with circumferential tissue capture, achieving immediate endoscopic closure of the defect.

**Figure 3:**
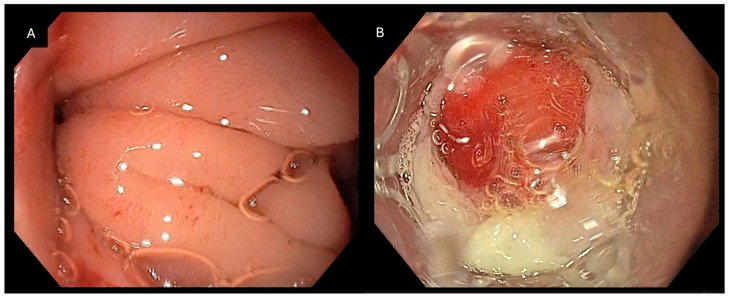
Endoscopic management of a duodenoperitoneal fistula following cholecystectomy: (**A**) Endoscopic view of a fistulous defect in the duodenal wall consistent with communication to the peritoneal cavity. (**B**) Deployment of the Padlock Clip resulting in circumferential tissue capture and immediate closure of the duodenal defect.

**Figure 4:**
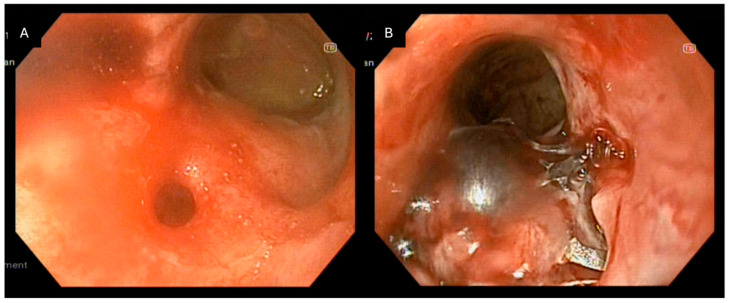
Endoscopic closure of a rectovaginal fistula following low anterior rectal resection for rectal cancer: (**A**) Endoscopic visualization of a fistulous opening in the rectal wall consistent with communication to the vaginal cavity. (**B**) Deployment of the Padlock Clip achieving circumferential approximation of the fistulous edges and immediate closure of the defect.

Regarding the type of communication, the most common presentation was esophagopleural or esophagoperitoneal communication, identified in 13 patients (46.4%). Other patterns included esophagotracheal or esophagobronchial fistulas (*n* = 4, 14.3%), duodenoperitoneal fistulas (*n* = 3, 10.3%), gastrocutaneous fistulas (*n* = 2, 7.1%), rectovaginal fistulas (*n* = 2, 7.1%), and other less frequent communications (*n* = 4, 14.3%) (Table 2; Figure 1, Figure 2, Figure 3 and Figure 4).

### 3.3. Procedural Characteristics of Endoscopic Treatment

Most fistulas were managed with a single Padlock Clip (*n* = 21, 75.0%), whereas two clips were required in five cases (17.9%) and three clips in two patients (7.1%).

The Standard Padlock system (9.5–11 mm) was used in the majority of cases (*n* = 25, 89.3%), while the Pro-Select system (11.3–14 mm) was applied in three patients (10.7%), primarily in larger or thicker-walled defects.

**Figure 5:**
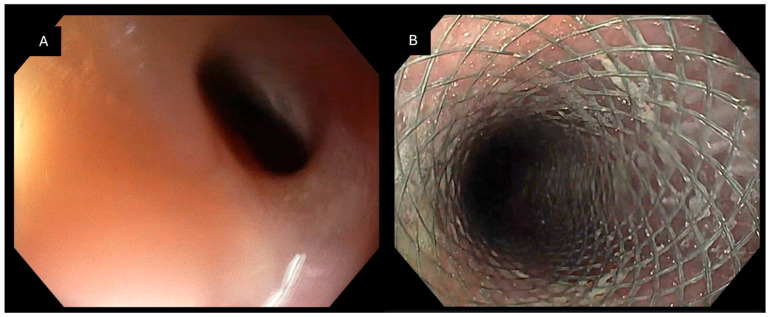
Postoperative fistula following gastrectomy for gastrointestinal stromal tumor (GIST): (**A**) Endoscopic visualization of a postoperative fistulous defect after gastrectomy performed for GIST. (**B**) Initial management with placement of a self-expanding metal stent (SEMS), which fails to achieve definitive closure, highlighting the therapeutic challenges associated with postoperative leaks.

**Figure 6:**
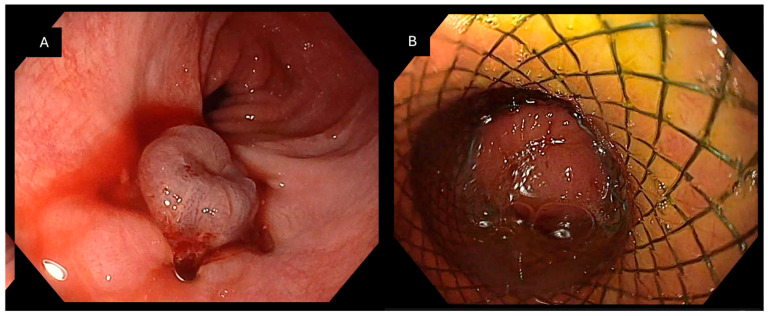
Multimodal endoscopic management of an esophagoperitoneal fistula following sleeve gastrectomy: (**A**) Initial closure of a distal esophageal fistula communicating with the peritoneal cavity using the Padlock Clip, demonstrating adequate circumferential tissue capture. (**B**) Placement of a self-expanding metal stent (SEMS) due to persistent fistula during follow-up, illustrating the need for adjunctive therapy in selected complex cases.

Tissue preparation was rarely required, with thermal edge preparation performed in only one case (3.6%). Adjunctive therapies were used in 11 patients (39.3%), either as part of a planned multimodal closure strategy in selected complex fistulas or as rescue therapy following incomplete closure or persistent leakage during follow-up. The most frequently applied adjunctive therapy was self-expanding metal stent (SEMS, Figure 5 and Figure 6) placement (*n* = 8, 28.6%), followed by additional clipping devices (Lockado^®^, *n* = 2, 7.1%) and tissue adhesive application (Glubran^®^, *n* = 1, 3.6%) (Table 3).

### 3.4. Clinical Outcomes and Safety Results

Technical success, defined as successful deployment of the Padlock Clip™ with immediate endoscopic closure of the fistula, was achieved in 25 of 28 patients (89.3%; 95% CI 0.72–0.98). Clinical success was defined as sustained fistula closure without surgical intervention regardless of whether additional endoscopic therapies were required during follow-up. This definition reflects real-world multimodal endoscopic management rather than standalone device efficacy. Clinical success was observed in 23 patients (82.1%; 95% CI 0.63–0.94). Adjunctive endoscopic therapies were used in a subset of patients either as part of a planned multimodal strategy or as rescue treatment following incomplete initial closure; therefore, the reported clinical success rate reflects final fistula closure outcomes within a multimodal treatment framework rather than standalone clip-only efficacy. The median clinical follow-up duration was 6.5 months (range 3–32 months).

Persistent fistulas were identified in five patients (17.9%), and reintervention was required in seven patients (25.0%; 95% CI 0.11–0.45). Reinterventions were performed in patients with persistent fistula or incomplete closure identified during follow-up evaluation. In most cases, reintervention was guided by clinical symptoms, radiological or endoscopic evidence of persistent fistula communication after the index procedure. Additional endoscopic management strategies included self-expanding metal stent placement and repeat clipping procedures according to defect characteristics and clinical evolution.

Adverse events occurred in three patients (10.7%; 95% CI 0.02–0.28) and consisted exclusively of clip migration. Clip migration was classified as an adverse event according to standard safety reporting conventions due to loss of device position and therapeutic function, despite absence of bleeding, perforation, procedure-related mortality, or other serious complications. All migration events occurred in the sleeve gastrectomy subgroup, consistent with the observed trend toward higher complication rates in this anatomically challenging location (Table 4).

### 3.5. Exploratory Subgroup Analysis

#### 3.5.1. Sleeve vs. Non-Sleeve Groups

An exploratory comparison between sleeve gastrectomy-related fistulas and non-sleeve cases was performed.

Technical success was achieved in 8 of 11 sleeve gastrectomy patients (72.7%; 95% CI 43.4–90.3%) compared with 17 of 17 non-sleeve patients (100%; 95% CI 81.6–100%). This difference approached statistical significance (Fisher’s exact test, *p* = 0.050), suggesting a possible trend toward lower technical success in sleeve gastrectomy-related fistulas.

Clinical success was observed in 72.7% of sleeve cases (95% CI 43.4–90.3%) and 88.2% of non-sleeve cases (95% CI 65.7–96.7%), without a statistically significant difference (*p* = 0.35).

Reintervention rates were comparable between groups (18.2% vs. 29.4%, *p* = 0.67).

Adverse events occurred exclusively in sleeve gastrectomy-related fistulas (27.3%; 95% CI 9.7–56.6%), whereas no complications were observed in non-sleeve cases (*p* = 0.050).

Given the small sample size, these findings should be interpreted cautiously and considered hypothesis-generating.

#### 3.5.2. Analysis of Factors Associated with Persistent Fistulas

An exploratory comparison between patients achieving clinical healing (*n* = 23) and those with persistent fistula (*n* = 5) was performed.

Technical success was associated with a higher likelihood of clinical healing (OR 10.71; 95% CI 1.06–108.64), with borderline statistical significance (Fisher’s exact test, *p* = 0.073), reflecting limited statistical power.

Use of a single clip showed a trend toward improved clinical outcomes (OR 6.07; 95% CI 0.89–41.36; *p* = 0.082).

Sleeve gastrectomy etiology, adjunctive therapy use, and upper gastrointestinal location were not significantly associated with persistent fistula.

These findings should be interpreted cautiously given the limited sample size and exploratory design.

#### 3.5.3. Outcomes Stratified by Fistula Complexity

Patients were stratified into simple (*n* = 11) and complex (*n* = 17) fistulas according to predefined criteria. Complex fistulas were defined as those requiring more than one clip, adjunctive therapy, or measuring ≥ 15 mm.

Technical success was achieved in all simple fistulas (100%) compared with 82.4% in complex cases (OR 5.55; 95% CI 0.26–118.72; *p* = 0.258). Clinical healing occurred in 90.9% of simple cases versus 76.5% of complex cases (OR 2.33; 95% CI 0.31–17.52; *p* = 0.619).

Reintervention was required in 9.1% of simple fistulas compared with 35.3% of complex fistulas (OR 0.25; 95% CI 0.04–1.80; *p* = 0.191). Notably, all adverse events occurred in the complex group (Table 5).

Although statistical significance was not reached, consistent trends toward improved outcomes in simple fistulas were observed.

## 4. Discussion

In this single-center experience, the Padlock Clip™ demonstrated high technical success (89.3%) and favorable clinical healing rates (82.1%) in the management of post-surgical gastrointestinal fistulas involving predominantly upper gastrointestinal locations. These results should be interpreted within the context of multimodal endoscopic management strategies. Adjunctive therapies were required in a subset of patients to achieve sustained fistula closure. Reintervention was required in 25% of cases, and adverse events were infrequent and limited to clip migration. These findings support the feasibility and safety of the Padlock system in a real-world cohort and are consistent with previously reported technical success rates ranging from approximately 80% to 95% for over-the-scope clip (OTSC) devices used in gastrointestinal defect closure [12,13,21]. Published experience with the Padlock Clip™ remains limited but includes small case series describing successful closure of gastrointestinal defects such as perforations, leaks, and fistulas with technical success rates approaching 80–100% in selected cohorts [2,12]. Our results are consistent with these observations and further extend available evidence by demonstrating favorable outcomes across heterogeneous postoperative gastrointestinal fistulas treated in a real-world clinical setting.

Endoscopic closure of post-surgical fistulas remains technically challenging and is influenced by multiple factors, including defect size, chronicity, anatomical location, and tissue characteristics. Early endoscopic intervention after fistula detection is generally associated with improved closure rates in previous studies [22]. The inclusion of both acute and chronic fistulas reflects the heterogeneity encountered in routine clinical practice; however, this may influence closure success rates and limit stratified interpretation of treatment response according to fistula chronicity. Chronic fistulas with fibrotic or epithelialized margins are generally less responsive to mechanical closure techniques and more frequently require multimodal endoscopic management strategies [11,14]. The absence of a standardized time-based classification limited subgroup analysis according to fistula chronicity and should be considered when interpreting the present results. Classification into acute versus chronic fistulas relied partly on endoscopic appearance and treatment history rather than predefined time-based thresholds, which may limit reproducibility of chronicity stratification. Furthermore, complete timing data between index surgery and endoscopic intervention were not consistently available, preventing detailed stratified outcome analysis according to fistula chronicity within the present cohort. A recent multicenter study confirmed the effectiveness of OTSC systems in the management of postoperative fistulas while emphasizing the importance of appropriate patient selection and adjunctive therapies to optimize outcomes [20,23]. Our technical and clinical success rates fall within these reported ranges, reinforcing the role of circumferential clipping devices such as the Padlock Clip as effective minimally invasive treatment options.

In our exploratory subgroup analysis, fistulas occurring after sleeve gastrectomy demonstrated lower technical success compared with those arising from other surgical procedures. This observation aligns with previous studies reporting that fistulas following sleeve gastrectomy, particularly near the esophagogastric junction, represent a challenging clinical entity due to increased intraluminal pressure, compromised vascularization, and mechanical stress at this anatomical site [5,6,24]. These physiological and anatomical factors may impair healing and contribute to higher rates of persistence or the need for additional interventions. Given the distinct anatomical characteristics and technical challenges associated with fistulas after sleeve gastrectomy, larger dedicated studies focusing specifically on this subgroup are warranted to further clarify optimal endoscopic management strategies.

Immediate technical success was associated with a higher likelihood of clinical healing in exploratory analysis, although this did not reach statistical significance and should be interpreted cautiously given the limited sample size. This observation aligns with previous studies suggesting that complete closure of gastrointestinal defects during the index endoscopic procedure may improve the likelihood of durable healing [7,12]. Adequate tissue capture and appropriate device selection are therefore critical to optimize therapeutic outcomes.

To further explore outcome determinants, we introduced a predefined classification of fistula complexity. Complex fistulas (defined by larger size, need for adjunctive therapy, or multiple clip deployment) demonstrated lower healing rates, higher reintervention frequency, and all observed adverse events. These findings are consistent with prior reports indicating that larger and more complex defects are less likely to respond to single-modality endoscopic closure and often require multimodal approaches combining clipping, stenting, or other techniques [11,25,26].

**Figure 7:**
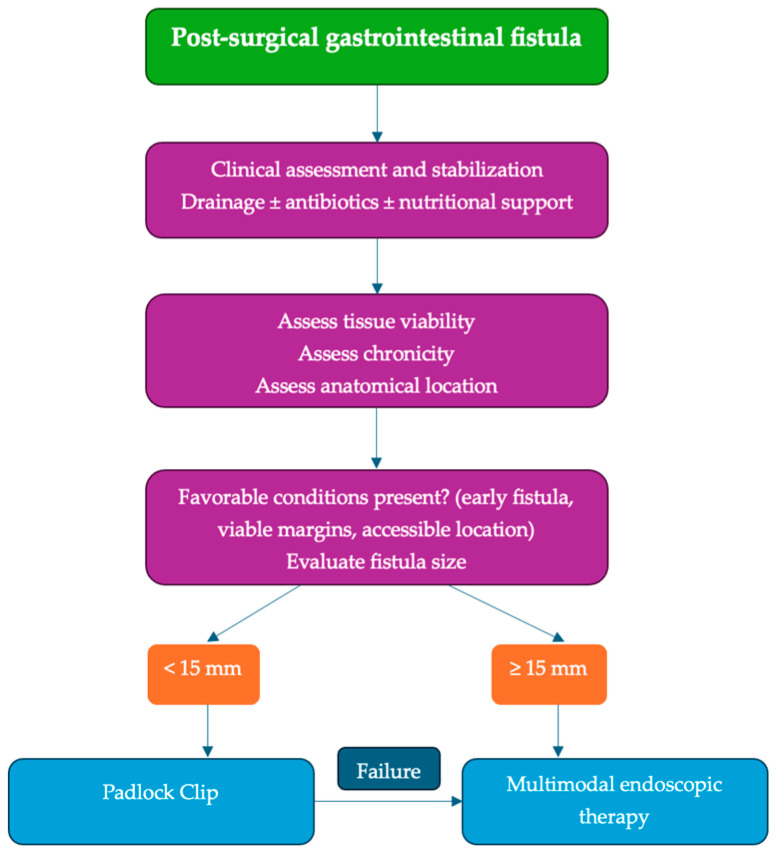
Practical considerations for Padlock Clip deployment within multimodal endoscopic management of post-surgical gastrointestinal fistulas based on cohort experience and the available literature.

Adjunctive endoscopic therapies were required in approximately 40% of our patients, most commonly self-expanding metal stent placement. This reflects the heterogeneous nature of postoperative fistulas and supports the concept that clipping devices frequently represent one component of a broader endoscopic treatment strategy rather than a standalone solution in all cases [25,26,27]. Multimodal endoscopic management has been increasingly recognized as an effective approach in complex or persistent fistulas. Based on these observations, a schematic overview summarizing practical treatment considerations for Padlock Clip deployment within multimodal endoscopic management strategies is presented in Figure 7. 

The Padlock Clip represents a newer OTSC device with a circumferential nitinol design that provides uniform tissue compression while preserving the endoscope working channel. Early clinical reports and small case series have demonstrated its feasibility and safety in the closure of gastrointestinal defects, including fistulas and perforations [2,16,23]. Our study contributes additional real-world data demonstrating favorable technical and clinical outcomes across a spectrum of postoperative fistulas involving both upper and lower gastrointestinal locations.

Although formal cost-effectiveness analysis was beyond the scope of the present study, minimally invasive endoscopic closure strategies such as Padlock Clip deployment may reduce the need for surgical reintervention and prolonged hospitalization in selected patients. Future prospective studies evaluating resource utilization and economic impact of circumferential clipping devices in the management of postoperative gastrointestinal fistulas would be valuable.

This study has several limitations. First, its retrospective and single-center design may limit generalizability and introduce potential selection bias. Accordingly, the present findings should be interpreted primarily as real-world feasibility and outcome data rather than comparative effectiveness evidence applicable to broader patient populations. Second, the relatively small sample size limits statistical power, particularly in subgroup analyses, and results should therefore be interpreted cautiously. The limited sample size also precluded multivariate regression analysis to identify independent predictors of treatment success or failure, and therefore observed associations should be interpreted as exploratory. In addition, the predominance of fistulas measuring <15 mm in our cohort may have contributed to the relatively high closure rates observed and should be considered when interpreting the effectiveness of clipping strategies in larger or more complex defects. Third, the heterogeneity of fistula etiologies, anatomical locations, and adjunctive therapies may influence outcomes. Finally, the absence of a control group prevents direct comparison with alternative endoscopic or surgical treatment modalities. Therefore, the present study evaluates feasibility and clinical outcomes of Padlock Clip deployment within routine clinical practice rather than relative efficacy compared with alternative endoscopic closure techniques such as stent placement or other OTSC systems. In addition, the median follow-up duration of 6.5 months may limit assessment of the long-term durability of fistula closure. Although the majority of patients had at least 6 months of follow-up, longer-term durability beyond 12 months was available only in a subset of cases and therefore late recurrence after apparently successful closure cannot be completely excluded. Moreover, confirmation of fistula closure was based on clinical and endoscopic follow-up depending on availability, which may introduce variability in outcome assessment. Because follow-up evaluation was performed according to clinical evolution rather than predefined protocol time points, the interval between index Padlock Clip deployment and reintervention varied among patients. However, reinterventions were performed within the expected follow-up period used for assessment of endoscopic fistula closure in routine clinical practice. Furthermore, the interval between index surgery and Padlock Clip placement represents an important determinant of fistula closure success; however, this variable was not consistently available due to referral-based patient pathways and the retrospective study design, which limited detailed stratified analysis according to timing of intervention.

Despite these limitations, our findings provide meaningful real-world evidence supporting the safety and effectiveness of the Padlock Clip in the management of post-surgical gastrointestinal fistulas and highlight the importance of achieving immediate technical success and appropriate patient selection to optimize clinical outcomes.

## 5. Conclusions

In this cohort, the Padlock Clip represents a safe and effective endoscopic treatment option for post-surgical gastrointestinal fistulas. Immediate technical success appears to be an important determinant of durable fistula closure and may help guide therapeutic decision-making and optimize patient selection. Complex fistulas and sleeve gastrectomy-related defects may require multimodal endoscopic management strategies. Larger prospective studies are needed to further define optimal patient selection and therapeutic algorithms.

## Figures and Tables

**Table 1:** Baseline and surgical background.

Variable	*n* (%)
Sex	
Female	17 (60.7%)
Male sex	11 (39.3%)
Underlying pathology leading to index surgery	
Malignant condition	10 (35.7%)
Gastrointestinal malignancy	8 (80.0%)
Extraintestinal malignancy	2 (20.0%)
Benign condition	18 (64.3%)
Morbid obesity	11 (61.1%)
Others	7 (38.9%)
Primary surgical procedure	
Gastric surgery	16 (57.1%)
Sleeve gastrectomy	11 (39.3%)
Other gastrectomy	5 (17.9%)
Abscess drainage	3 (10.7%)
Low anterior rectal resection	2 (7.1%)
Pulmonary lobectomy	2 (7.1%)
Others *	5 (17.6%)

* Others include transduodenal excision of a duodenal neuroendocrine tumor with pyloro-plasty, Heller myotomy, cholecystectomy, and cytoreductive surgery for ovarian cancer involving colorectal resection.

**Table 2:** Fistula characteristics.

Variable	*n* (%)
GI segment	
Upper	25 (89.3%)
Lower	3 (10.7%)
Anatomical location	
Esophagus	10 (35.7%)
Esophagogastric junction	5 (17.9%)
Esophagojejunal anastomosis	3 (10.7%)
Stomach	3 (10.7%)
Duodenum	4 (14.3%)
Rectum	3 (10.7%)
Fistula size	
<15 mm	26 (92.9%)
≥15 mm	2 (7.1%)
Type of communication	
Esophagopleural/Esophagoperitoneal	13 (46.4%)
Esophagotracheal/Esophagobronchial	4 (14.3%)
Gastrocutaneous	2 (7.1%)
Duodenoperitoneal	3 (10.7%)
Rectovaginal	2 (7.1%)
Others *	4 (14.3%)

* Others include rare or isolated communications not fitting predefined categories.

**Table 3:** Endoscopic procedure characteristics.

Variable	*n* (%)
Number of clips	
1	21 (75.0%)
2	5 (17.9%)
3	2 (7.1%)
Clip size	
Standard (9.5–11 mm)	25 (89.3%)
Pro Select (11.3–14 mm)	3 (10.7%)
Tissue preparation	
None	27 (96.4%)
Thermal edge preparation	1 (3.6%)
Adjunctive therapy	
None	17 (60.7%)
Self-expanding metal stent (SEMS)	8 (28.6%)
Additional clipping device	2 (7.1%)
Tissue adhesive	1 (3.6%)

**Table 4:** Outcomes and safety.

Variable	*n* (%)
Technical success	
Yes	25 (89.3%)
No	3 (10.7%)
Reintervention	
Yes	7 (25.0%)
No	21 (75.0%)
Clinical success	
Yes (healing fistula)	23 (82.1%)
No (persistent fistula)	5 (17.9%)
Adverse events	
No	25 (89.3%)
Yes	3 (10.7%)
Clip migration	3 (100% of adverse events)

**Table 5:** Outcomes according to fistula complexity.

Outcome	Simple (*n* = 11)	Complex (*n* = 17)	OR (95% CI)	*p*-Value
Technical success	11 (100%)	14 (82.4%)	5.55 (0.26–118.72)	0.258
Clinical success	10 (90.9%)	13 (76.5%)	2.33 (0.31–17.52)	0.619
Reintervention	1 (9.1%)	6 (35.3%)	0.25 (0.04–1.80)	0.191
Adverse events	0 (0%)	3 (17.6%)	0.18 (0.01–3.85)	0.258

## Data Availability

Data that support the findings of this study and materials are available from the first author upon reasonable request.

## Abbreviations

The following abbreviations are used in this manuscript:

CI	Confidence Interval
EGJ	Esophagogastric Junction
GI	Gastrointestinal
GIST	Gastrointestinal Stromal Tumor
IRB	Institutional Review Board
OR	Odds Ratio
OTSC	Over-the-Scope Clip
SEMS	Self-Expanding Metal Stent
